# IKAROS in Acute Leukemia: A Positive Influencer or a Mean Hater?

**DOI:** 10.3390/ijms24043282

**Published:** 2023-02-07

**Authors:** Maria Rosa Conserva, Immacolata Redavid, Luisa Anelli, Antonella Zagaria, Francesco Tarantini, Cosimo Cumbo, Giuseppina Tota, Elisa Parciante, Nicoletta Coccaro, Crescenzio Francesco Minervini, Angela Minervini, Giorgina Specchia, Pellegrino Musto, Francesco Albano

**Affiliations:** 1 Hematology Section, Department of Precision and Regenerative Medicine and Ionian Area (DiMePRe-J), University of Bari ‘Aldo Moro’, 70124 Bari, Italy; 2 School of Medicine, University of Bari ‘Aldo Moro’, 70124 Bari, Italy

**Keywords:** *IKZF1*, acute lymphoblastic leukemia, acute myeloid leukemia, molecular pathways, leukemogenesis

## Abstract

One key process that controls leukemogenesis is the regulation of oncogenic gene expression by transcription factors acting as tumor suppressors. Understanding this intricate mechanism is crucial to elucidating leukemia pathophysiology and discovering new targeted treatments. In this review, we make a brief overview of the physiological role of IKAROS and the molecular pathway that contributes to acute leukemia pathogenesis through *IKZF1* gene lesions. IKAROS is a zinc finger transcription factor of the Krüppel family that acts as the main character during hematopoiesis and leukemogenesis. It can activate or repress tumor suppressors or oncogenes, regulating the survival and proliferation of leukemic cells. More than 70% of Ph+ and Ph-like cases of acute lymphoblastic leukemia exhibit *IKZF1* gene variants, which are linked to worse treatment outcomes in both childhood and adult B-cell precursor acute lymphoblastic leukemia. In the last few years, much evidence supporting IKAROS involvement in myeloid differentiation has been reported, suggesting that loss of *IKZF1* might also be a determinant of oncogenesis in acute myeloid leukemia. Considering the complicated “social” network that IKAROS manages in hematopoietic cells, we aim to focus on its involvement and the numerous alterations of molecular pathways it can support in acute leukemias.

## 1. Introduction

IKAROS, encoded by the *IKZF1* (Ikaros family zinc finger 1) gene, is a transcription factor that is crucial for lymphocyte specification and differentiation. Its role is pivotal for all hematopoietic cell types, from stem cells to mature lymphoid and myeloid cells. IKAROS is essential for normal hematopoiesis, autoimmunity and tumor suppression. *IKZF1* variants were associated with lymphoblastic cell deficiency, autoimmunity and the development of malignancies, including hematological diseases, particularly acute leukemia (AL) [1]. IKAROS is well known to be involved in different ways in the pathogenesis of acute lymphoblastic leukemia (ALL) [2], and its role also in the context of acute myeloid leukemia (AML) is increasingly emerging [3]. In this review, our focus is to unravel the intricate social network of IKAROS, which is involved in numerous interactions with several proteins as well as in many relationships with the main characters in cellular pathways. In particular, we consider its involvement in leukemogenesis, highlighting its two-faced role as both tumor suppressor and oncogenic factor, starting with its biological profile.

## 2. Genetic, Expression and Post-Translational Modification: A “Bio” of IKAROS

### 2.1. Genetic and Structure of IKZF1

**Figure 1:**
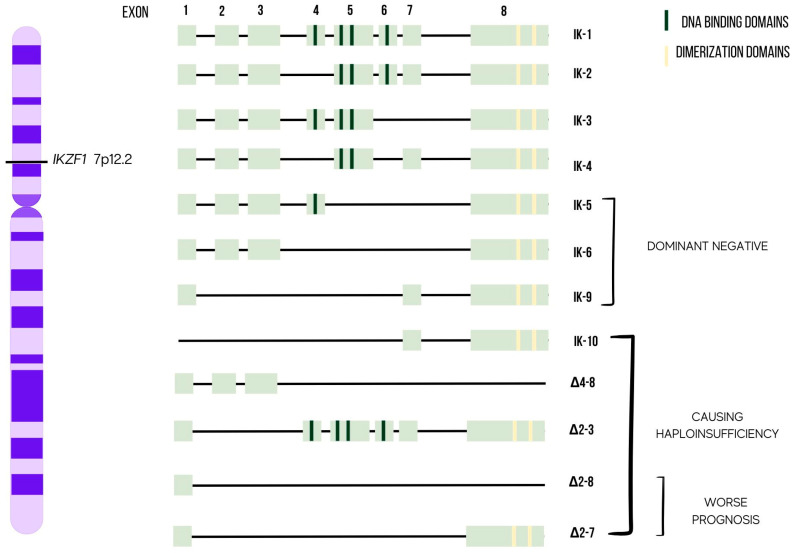
Graphic representation of IKAROS isoforms. The N-terminal zinc fingers are shown in dark green bars and C-terminal zinc fingers are shown in yellow bars. Isoforms from IK-5 to IK-9 are dominant negative (DN) [10], isoforms IK-10, Δ4-8, Δ2-3, Δ2-8, Δ2-7 cause haploinsufficiency [11,12,13,14,15].

IKAROS is a zinc finger transcription factor encoded by the *IKZF1* gene that was first discovered in the early 1990s by the Smale and Georgopoulos groups [4,5,6]. The members of the IKAROS zinc finger protein family belong to the Krüppel family and consist of other transcription factors: HELIOS, AIOLOS, EOS, and PEGASUS (encoded respectively by *IKZF2-5*) [7]. These transcription factors are essential in regulating normal lymphopoiesis, but they also have pleiotropic functions in almost all hematopoietic cell types, from stem cells to mature lymphoid and myeloid cells. The *IKZF1* gene is mapped on chromosome band 7p12.2, consists of 8 exons and encodes 519 amino acids (Figure 1). It encodes at least 16 isoforms (IK1-IK16) generated by alternative splicing, which are subdivided into “DNA-binding” (e.g., IK-1) and “non-DNA-binding” (e.g., IK-6) isoforms, depending on the presence or absence of N-terminal zinc finger (ZF) domains [6]. All the isoforms can contain up to six ZF modules, which are structured into two domains: the conserved N-terminal DNA-binding domain (DBD), including up to four ZF motifs, conferring the ability to bind to the core GGGAA motif (except PEGASUS); and the C-terminus, containing two ZF motifs, required for protein-protein interaction, that is crucial for IKAROS activity [6]. IKAROS dimerization can occur either with other HELIOS, AIOLOS, or EOS isoforms (heterodimerization) or with its alternative splicing isoforms (homodimerization), which exponentially increases the possible combination rates. These interactions may enhance or suppress the affinity of DNA-binding, thereby impacting IKAROS transcription activity [8,9].

IKAROS can bind target genes as multimers and monomers as well as dimers. IKAROS activating or inhibiting effects on the genome are caused by a complicated combinatory impact of homo-/hetero-/di-/multimerization between DNA-binding and non-DNA-binding isoforms [16]. It has also been suggested that IKAROS dimers may form higher-order complexes containing multiple DBDs that may mediate interactions among distal sites, supporting communication between distal regulatory elements [6]. In addition, alternative combinations of ZF modules impact the capability of DNA-binding and functional properties of the several isoforms and, consequently, their effects on transcription [16]. The variable number of DBDs confers a different functional property and subcellular localization to IKAROS isoforms: in fact, those containing at least three DBDs are capable of binding DNA sites; on the contrary, isoforms with less than three DBDs remain in the cytoplasm [6]. Only IK-1, IK-2 and IK-3 have an appropriate N-terminal DBD suitable for high-affinity DNA, while all the isoforms share the two C-terminal motifs. Furthermore, DNA affinity is increased by homo- and heterodimer interactions among the DNA-binding isoforms such as IK-1, IK-2 and IK-3 [17]; on the contrary, heterodimers between isoforms with and without DBDs are transcriptionally ineffective [17]. Thus, IKAROS proteins with fewer than three N-terminal ZFs can play a dominant negative (DN) role in transcription by interfering with the activity of isoforms that bind DNA. Via these mechanisms, in which a wide range of genes are involved in the development of myeloid cells, as well as in the positive regulation of lymphocyte differentiation and the negative regulation of cell proliferation, peripheral lymphocyte homeostasis can be activated [7,18,19,20,21]. 

Besides DBDs and dimerization domains, all the isoforms share a conserved bipartite activation domain adjacent to the C-terminal ZF, consisting of two functionally distinct acid and hydrophobic regions. These two subdomains are a strong activation module able to stimulate basal levels of transcription activation of IKAROS target genes [8]. 

### 2.2. Expression of IKZF1

The pattern of expression of *IKZF1* in mice, broadly dispersed within the embryonic and adult hematopoietic systems, provided the first hints about its function. In fact, it has been seen that Ikaros is more prevalent in developing thymocytes, mature T, B, and natural killer cells, as well as embryonic hematopoietic progenitors, while it is less prevalent in erythroid and myeloid precursors [8]. This restricted and complex expression pattern in embryonic, fetal, and adult hematopoietic sites identifies this gene as a possible regulator of cell fate in the fetal and adult hematopoietic systems. IKAROS isoforms also exhibit various expression patterns, with IK-1 and -2 being the most prevalent throughout lymphocyte development, IK-3, -5, and -6 less prevalent, and IK-4 only seen in early T-cell progenitors [16]. 

### 2.3. Post-Translational Modification of IKAROS

Many functional processes, such as methylation, transcript level and post-translational modifications, may modulate the function of IKAROS. It has been observed that hypermethylation of CpG islands in IKAROS reduces its expression, while multiple phosphorylations by casein kinase II (CK2) and protein phosphatase 1 (PP1) affect its activity [22], modulating its DNA-binding affinity, subcellular localization and stability [23]. CK2 directly phosphorylates IKAROS at multiple sites during mitosis, and this hyperphosphorylation promotes its degradation by the ubiquitin/proteasome pathway [22]. As a consequence, IKAROS is a target of immunomodulatory drugs promoting proteasomal degradation [24]. The capacity of IKAROS to control cell cycle progression at the G1/S checkpoint is compromised by CK2-mediated phosphorylation of these amino acids [25]. It has been reported that PP1 dephosphorylation, in contrast to CK2, increases IKAROS stability and its DNA-binding affinity and re-establishes its pericentromeric localization [22]. CK2 and PP1 balance this phosphorylation state of IKAROS in normal thymocytes, so acute leukemia may arise if this equilibrium is disrupted. The IKAROS repression function is compromised by SUMOylating of its lysine residues [26], while its nuclear localization and DNA-binding activity is enhanced by the phosphorylation of specific serine residues t [27]. 

## 3. IKAROS Is “LinkedIn” in Many Biological Pathways

IKAROS’ essential function in controlling hematopoiesis has been validated by many mouse gene targeting studies. DNA sequence analysis [16] or the examination of expression patterns in gene targeting studies [28,29,30] have been used to identify putative gene targets that IKAROS may control. The majority of them are hematopoietic-specific, including the *TCR* and *CD3* genes [16]. 

It is well known that IKAROS plays a significant and wide-ranging role in biology, most likely employing a combination of methods to control transcription, depending on the kind of cell and stage of development. It appears to operate both as a transcriptional repressor and as an activator through its ability to bind to different nuclear factors involved in epigenetic regulation and chromatin remodeling. This flexibility is made possible, in part, by the variety of proteins with which IKAROS can interact, such as the co-repressor CtBP, the viral oncoprotein E1A, the histone deacetylase repressor complexes NURD and SIN3, Polycomb repressive complex 2 (causing gene repression), as well as the nucleosome remodeling complex SWI/SNF (resulting in gene activation), in addition to other family members. 

IKAROS can regulate expression of its target genes both directly, by binding to their promoters, as well as by altering the global epigenetic signature of enhancer and super-enhancer landscapes [31,32]. Because of the characteristics of these binding partners, chromatin reorganization is thought to play a major role in how IKAROS affects transcription. It has been seen that, in activated lymphocytes, IKAROS is localized in regions of pericentromeric heterochromatin areas; in this way, it may directly induce heterochromatic silencing by targeting the NURD complex via Mi-2 [28]. Additionally, it has been proposed that IKAROS may also potentiate gene expression in cycling cells but it cannot activate transcription by itself; instead, IKAROS may increase gene expression, maintaining repression complexes distant from the promoters and promoting access to transcriptional activators [33]. It has also been shown that IKAROS primarily controls gene expression through its connection with the nucleosome remodeling and deacetylase complex, including the ATP-dependent chromatin remodeling proteins CHD3 and CHD4 and the histone deacetylases HDAC1, HDAC2, and HDAC1 [34]. Additionally, IKAROS may be directly involved in initiating transcription, interacting with general transcription factors, such as TFIIB and TBP [35]. Furthermore, IKAROS is involved in transcription elongation control because of its transfer mediated by PP1 to CDK9, which promotes the activation of P-TEFb, facilitating transcription elongation of IKAROS target genes in hematopoietic cells [36]. 

### 3.1. Lymphoid Landscape

Several studies have suggested that the IKAROS family is required at different stages of lymphocyte development [37], being involved especially in cellular processes like proliferation, differentiation, cell cycle arrest, and apoptosis [38]. However, it is also crucial for normal myeloid, megakaryocyte and erythroid differentiation [30]. 

The role of the IKAROS family members in the differentiation and properties of single T helper cell subsets, including Th1, Th2, Th17, T follicular, and Tregs, has been amply studied [39]. Several studies have revealed that IKAROS promotes Th17 and Treg cell differentiation and suppresses the polarization of Th1 [40]. 

The pre-B-cell receptor (BCR) signal pathway is the most closely studied IKAROS-related pathway because it plays a crucial role in regulating the transcription of genes implicated in signaling, cell survival, stromal-cell adhesion, and B-cell commitment during pre-B-cell differentiation [41]. The activated pre-BCR signaling pathway results in the phosphorylation of FOXO1, which, when exported out of the nucleus, is subjected to proteasome degradation. The decreased expression level of FOXO1 leads to improper splicing of IKAROS mRNA [42], which in turn counteracts this by suppressing two sites in the pathway [43,44]. 

NOTCH is another well-studied IKAROS-related pathway that is crucial for tumor cell proliferation. Indeed, in a pre-T-cell receptor (TCR)-dependent mode, NOTCH3 upregulates the expression of RNA-binding protein HuD, switching the alternative splicing of IKAROS to a DN isoform [45]. Conversely, IKAROS counteracts the NOTCH effect on CSL activation, competing with its DNA-binding site, and represses the expression of downstream genes, including the component of pre-TCR [46]. 

Several studies suggested that IKAROS is a target of the MAPK signaling pathway as it was shown that ERK1/2 phosphorylation of ETS1 suppresses the ability to increase *IKZF1* expression. Studies have shown that the augmented ERK1/2 activity is due to activation of the integrin signaling pathway, which is reported to be limited by IKAROS [47]. 

Finally, it is shown to be influenced by several interferon regulatory factors (IRFs), including IRF4 and IRF8, that induce its expression, as well as AIOLOS expression [48], while IRF5 seems to act as an inhibitor [49]. 

Genome-wide studies [50] have reported thousands of IKAROS targets, including many downstream genes crucial for lymphocyte development, in particular *DNTT* [51] and the *RAG* locus [52] for *VDJ* recombination, *CD8α* [53], *CD3δ*, *IL2* [54], *AHR*, *RUNX1* [55] and *STAT4* [56] for T-cell differentiation, and c-*MYC* in B-cell differentiation [57]. IKAROS has various impacts on the development, reproduction, and differentiation of numerous varieties of innate or adaptive lymphocytes. In fact, its function has been reported to be pivotal for the conversion between the large and small pre-B stages [41] and for B-cell proliferation and differentiation maintenance via kinase-signaling cascades and chromatin protein 4 [58]. In particular, IKAROS is essential for pro- and pre-B2 cell differentiation, promoting heavy- and light-chain gene rearrangement, inducing *RAG1/2* gene expression, and controlling chromatin accessibility at the *IgH* and *IgK* loci [59], upregulating the expression of genes crucial for cell survival, metabolism, and BCR signaling, stromal-cell adhesion and B-cell commitment, including *EBF1*, *PAX5* and *FOXO1* [41]. On the contrary, studies have shown that IKAROS contrasts the expression of genes regulated by the IL-7/STAT5 pathway [59]. Thus, IKAROS is a promoter of B2 cell differentiation but a negative regulator of B1 cell development [60]. Furthermore, it has been reported to regulate the trans-differentiation of ILC3-ILC1/NK cells [61], and to support the downregulation of *RAG1/2* gene expression in CD4+ CD8+ positive thymocytes [62]. 

IKAROS is involved in pre-TCR checkpoint control and T-cell activation downstream of the IL-2 receptor pathway at different stages of the T-lineage [63]. Several studies have demonstrated IKAROS’ pivotal role in preventing autoimmunity, influencing BCR unresponsiveness and suppressing TLR signaling transduction [64]. 

### 3.2. Myeloid Landscape

Besides lymphoid genes, many hematopoietic genes were found to be regulated by IKAROS, such as *iNOS* in macrophages [65] and γ-*GLOBIN* [36] and *GATA1* in primary megakaryocytes [66], although its role in myeloid cell functioning has not been well clarified. A mouse model has shown that IKAROS silences specific pathways in the ordinary precursors of macrophage–monocyte evolution, contributing to the regulation of the early stage of neutrophil differentiation, whilst it seems to be optional for mature neutrophils. Indeed, *IKZF1* mutant mouse models have shown defects in myelopoiesis, such as impaired terminal granulocyte differentiation and defects in neutrophil survival and migration [20]. It has also been reported to have a role in regulating the expression of iNOS synthase downstream of lipopolysaccharide/interferon-γ stimulation in a macrophage cell line [65]. IKAROS impacts also on plasmacytoid dendritic cell (pDC) development at multiple stages [67], interacting with NOTCH pathway activation to maintain homeostasis of monocyte and dendritic progenitors and common dendritic progenitors, promoting pDC development by antagonizing TGFβ1 signaling [68]. All these data taken together suggest that IKAROS regulates differentiation and immune function in the myeloid lineages, as it does in the lymphoid lineages.

## 4. *IKZF1* Dysregulation in ALL: A Mean “Hater”

**Figure 2:**
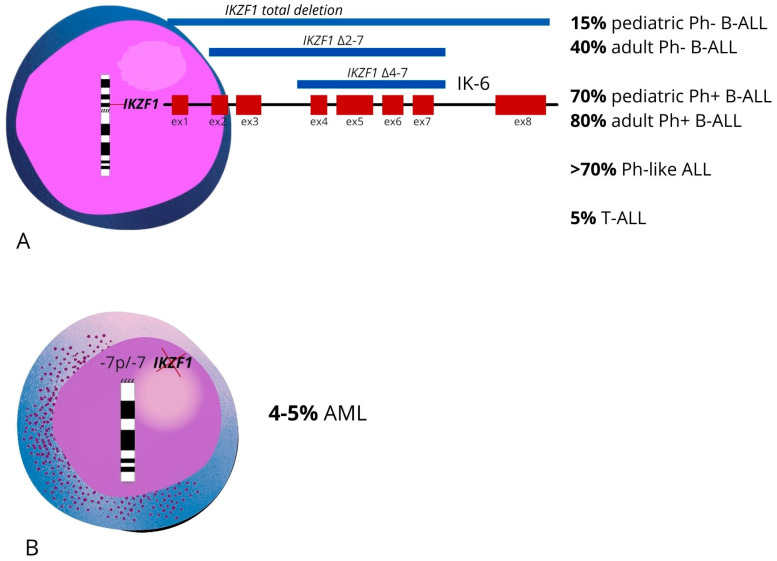
(**A**) Graphic representation of the frequency of the most common *IKZF1* alterations in acute lymphoblastic leukemia. (**B**) Graphic representation of the frequency of the most common *IKZF1* alterations in acute myeloid leukemia.

The Churchmann et al. study showed that *IKZF1* alterations cause cellular tumorigenicity, since it induces phenotypic alterations typical of leukemic cells, such as stem-cell-like properties, cell adhesion, cellular mislocalization and an impaired motility and response to hypoxia [69]. The spotlight has been on *IKZF1* since Mullighan et al. described, for the first time, the recurring, principally mono-allelic and focal deletion of the coding region of *IKZF1* in subjects affected by ALL [10,11]. The most recurrent deletions (Figure 2A) may involve the entire gene or part of it, and they are observed with an overall frequency of 15% in pediatric and 40% in adult Philadelphia-negative (Ph-) B-ALL cases [70,71]. *IKZF1* variants and deletions are more commonly identified in B-precursor ALL compared to T-precursor ALL. 

Considering that IKAROS is indispensable for B-cell commitment, several studies have shown that B-ALL patients with *IKZF1* abnormalities have a poor prognosis, a 3-fold increase in the risk of relapse after treatment [11,72] and a reduced 5-year event-free survival (EFS) of 61% compared to 87% for those without this abnormality [73]; in contrast, only 5% of patients with T-cell ALL harbor the loss of an IKAROS allele [2,74]. IKAROS alterations are closely associated with adult (about 80%) and pediatric Ph+ ALL (about 70%); their presence is associated with an adverse outcome [2,10,73,75,76]. Also, in Ph-like ALL, there is a close association with the presence of alterations of IKAROS. It has been reported that more than 70% of adults and pediatric Ph-like ALL patients are carriers of abnormalities in the *IKZF1* gene, resulting in a poor prognosis with significantly lower 5-year EFS rates compared to Ph-like ALL patients without an *IKZF1* alteration [77]. Furthermore, about 28 germline variants of *IKZF1* have been identified following the analysis of sporadic and familial pediatric B-ALL cases. The variants detected are distributed across the gene, even outside of known DNA-binding or dimerization domains and are mostly missense variants, to which two nonsense and one frameshift variant have been added. This fact suggests that *IKZF1* can be considered a predisposing gene for the onset of leukemia since, both in vivo and in vitro, these germline variants trigger leukemogenesis processes. Therefore, the germinal variants of *IKZF1* cause an alteration of normal lymphopoiesis and, hence, immunodeficiency and a predisposition to B-cell leukemia [78]. 

### 4.1. Deletions of IKZF1 Exons 

The loss of IKAROS function in most cases is due to deletions of exons coding for the DBDs, with the formation of DN isoforms that completely compromise the IKAROS function, but it cannot be excluded that post-anomalous transcription may alter its function [23,79]. The most detrimental isoforms of IKAROS are DN-related. In particular, DN IK-6 resulting from the deletion of exons 4–7 is considered highly oncogenic, and its presence is associated with a more severe phenotype [10]. Deletions involving exons 1–3 and 8, on the other hand, imply haploinsufficiency and, thus, reduced levels of IKAROS. This condition appears to be less “leukemogenic”, since mice with DN isoforms show a much more severe phenotype than mice with low levels of IKAROS [12,13,80]. The analysis of patients with Ph+ ALL described by Mullighan et al. showed that the most recurrent deletions are mono-allelic ones confined to exons 4–7 (Δ*IKZF1*), resulting in the presence of DN IK-6. However, other isoforms have also been identified, such as IK-9 and IK-10, with the deletion of exons 2–6 and exons 1–6, respectively [10]. In adult Ph+ ALL, it has been seen that 40–60% show Δ*IKZF1*, about 20% the deletion of exons 2–7, and just over 10% monosomy of 7. [70,81]. About 60% of children with B-ALL predominantly show total deletions of *IKZF1* and deletions of exons 4–7. A lower percentage of patients with deletions involving exons 2–3, exons 2–7, and exons 4–8 has been reported. In both Ph+ and Ph- ALL, only a minimal percentage of patients present a biallelic deletion of *IKZF1* [11,14]. Therefore, *IKZF1* distribution anomalies in ALL subgroups are not homogeneous, and each of them results in a different adverse outcome. Interestingly, deletion of exons 2–7 and exons 2–8 confers a worse prognosis than the more common deletions (whole gene deletion and Δ4–7) [15] (Figure 1). Depending on the alteration of *IKZF1,* the pathogenic effect may be different. Studies in mice have shown that a reduced expression of IKAROS tends to block differentiation at an early pro-B-cell stage, while the introduction of DN IK-6 in murine stem cells alters B-lymphocyte lineage commitment [82]. Δ*IKZF1*-mutated cases were shown to have a specific expression signature that involved the down-regulation of DNA-repair genes and up-regulation of stem-cell self-renewal and JAK–STAT signaling [11]. On the other hand, IK-6 expression is associated with treatment resistance and decreased apoptosis and increased proliferation [83].

### 4.2. Epistatic Effects of IKZF1 Deletion Impact All Patients’ Prognosis

Several studies have demonstrated that both focal and non-focal deletions of *IKZF1* confer a poor prognosis in terms of an increased risk of relapse and decreased EFS in most ALL subtypes. Studies on copy number variation (CNV) showed that specific combinations of co-occurring genetic copy number losses could have epistatic effects with a differential impact on treatment response. Δ*IKZF1* is rarely present (3–18%) in the prognostically favorable *ETV6::RUNX1* genetic subtype [14,84]. In contrast, the combined deletion of *BTG1* and *IKZF1* confers a worse 5-year EFS and a higher cumulative incidence of relapse than the *IKZF1* deletion alone [85]. Another epistatic effect has been established, in particular for *ERG* deletions, because the negative effect of an *IKZF1* deletion can be surprisingly mitigated when co-occurring with an *ERG* deletion. The co-presence of the two deletions results in a favorable outcome in B-ALL patients with an ERG deletion, with an 8-year EFS and overall survival (OS) of 86.4% and 95.6% respectively, even when associated with frequent *IKZF1* deletions [86]. Recently, a specific prognostic class called *IKZF1*^plus^ was introduced, proposed by the Associazione Italiana Ematologia and Oncologia Pediatrica–Berlin-Frankfurt-Muenster (AIEOP-BFM) study group. This classification is defined by the deletion of *IKZF1* with at least a co-occurring deletion in *CDKN2A/B, PAX5*, or the *PAR1* region in the absence of *ERG* loss [87]. In addition, the definition of *IKZF1*^plus^ excluded other frequent genomic losses affecting *ETV6*, *RB1*, *BTG1*, and *EBF1* since no significant impact on clinical outcome was observed [88]. *IKZF1*^plus^ confers an adverse prognosis compared to *IKZF1*^plus^-negative patients, with the EFS being 53% and 79%, respectively. Furthermore, *IKZF1*^plus^-positivity has been associated with severely increased risk of relapse, specifically in patients with detectable levels of minimal residual disease (MRD) after induction therapy [89]. It should be noted that neither constitutional nor acquired deletions of *IKZF1* seem to be sufficient to trigger the onset of leukemia [90]. However, it is argued that the co-presence of some genetic alterations can modulate, for better or worse, the pathogenic and prognostic impact of *IKZF1* alterations. *IKZF1* could be a secondary hit during leukemogenesis. This deletion is not often detected at the time of diagnosis, but often emerges at relapse, strongly indicating that *IKZF1* deletions are associated with treatment failure [10,73,80,91]. 

### 4.3. Other IKZF1 Alterations

Sequence variants at the level of the N-terminal domain of IKAROS have functional effects similar to those caused by focal deletions. Missense, nonsense and frameshift variants have been seen in a cohort of high-risk pediatric B-ALL patients [11,92,93]. Other studies focused on a subgroup of B-cell precursor (BCP) ALL patients with a distinct gene expression profile, including a point variant of *IKZF1* affecting the DBDs [94,95]. Gene fusions involving *IKZF1* are rare, although some have already been described in pediatric B-ALL. The most common partners are *NUTM1, STED5, CDK2,* and *TRPV2*,although their functional effects remain hypothetical [96]. A recent study by Rahmani et al. suggested that, in B-ALL patients, overexpression of *IKZF1* may contribute to B-cell differentiation arrest and proliferation induction. Therefore, when investigating the methylation status of CpG islands in the promoter region of *IKZF1* in B-ALL cells of children, a hypomethylation pattern of the gene promoter was observed in 96% of B-ALL samples [97].

## 5. Loss of Function of IKAROS in ALL: What’s Up? 

**Figure 3:**
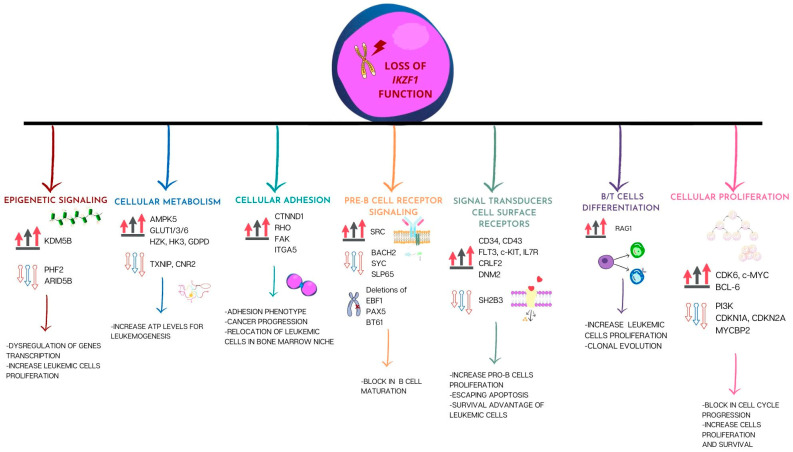
Graphical representation of the altered pathways (epigenetic signaling [100,101,102,103,104]; cellular metabolism [105]; cellular adhesion [106,107]; pre-B-cell receptor signaling [73,85,108,109]; signal transducer cell surface receptors [110,111,112,113,114]; B/T-cell differentiation [115]; cellular proliferation [110,116,117,118,119,120,121]) due to loss of *IKZF1* function in B-cell acute lymphoblastic leukemia.

IKAROS might activate or inhibit oncogenes or tumor suppressor genes; as a result, IKAROS-mediated transcriptional activation of target genes controls the survival and proliferative potential of cancer cells [98]. IKAROS is crucial for the healthy growth of the immune system, but too much of it has been associated with the development and spread of several cancers, particularly B-ALL [99] (Figure 3). *IKZF1* gene lesions that disrupt the role of lymphoid transcription factors influence the regulation of its target genes, whose activity is crucial for leukemia development and/or progression. 

### 5.1. Cellular Proliferation Pathways

IKAROS controls leukemia cell proliferation by inhibiting the gene transcription that promotes cell cycle progression and the phosphatidylinositol-3 kinase (PI3K) pathway. Song et al. [122] highlighted the relevance of IKAROS and how it controls leukemic cells’ ability to proliferate by preventing the transcription of the genes involved in the PI3K pathway. It has been shown that overexpression of IKAROS contributes to suppress the transcription of genes that promote the PI3K pathway, such as the oncogene *PIK3CD*; at the same time, it induces the transcription of the *INPP5D* gene that inhibits the PI3K pathway. Accordingly, the PI3K pathway was negatively regulated by IKAROS overexpression, decreasing the phosphorylation of AKT, a downstream target of the PI3K pathway [122]. It has been shown that IKAROS can modulate leukemic cell proliferation by inhibiting the transcription of genes involved in cell cycle progression, including *ANAPC1*, *ANAPC7*, *CDK2*, *CDK6*, *CDC2*, *CDC7*, *CDC16*, *CDC25c*, *CDC25a*, *CCND3*, and *CCNE2*, consequently resulting in a partial cell cycle arrest [31,122]. Loss of IKAROS function due to *IKZF1* gene deletions and variants affects cell proliferation through the repression of some targets, such as *CDKN1A* e *CDKN2A* [110], and the upregulation of other target genes, including *CDK6*, *c-MYC* and *BCL-6* [111,112]. Overexpression of the well-known oncogene *c-MYC* has been linked to various cancers, including leukemia and lymphoma. In adult ALL patients, IKAROS positively regulates the transcription of *MYCBP2* but represses the transcription of the *c-MYC* gene, and sequence analysis of the promoter regions of both *c-MYC* and *MYCBP2* revealed strong IKAROS-binding sites. Indeed, adult ALL patients with *IKZF1* haploinsufficiency displayed high c-MYC and decreased MYCBP2 concentrations [111]. Over 500 genes, mostly those involved in the cell cycle, gene transcription, tolerance to DNA damage, and control of chromatin structure, are affected by BCL6, a zinc finger transcriptional repressor [113]. Recent investigations revealed that increased BCL6 expression in adult B-ALL patients is related to *IKZF1* deletion [112]. Indeed, pre-B-cell survival and the preservation and protection of leukemia stem cells are regulated by an enhanced expression of BCL6 [114]. The increased BCL6 expression in ALL cells makes them more resistant to DNA deterioration, which improves survival when BCR-ABL1 kinase inhibition is applied [123]. 

### 5.2. Cell Commitment: Pre-B-Cell Receptor Signaling and B/T-Cell Differentiation

BCL6 orchestrates BACH2 protein stability in leukemia and lymphoma, and the BCL6/BACH2 axis equilibrium is crucial for controlling pre-BCR checkpoint cascades [124]. Furthermore, *BACH2* is a transcriptional factor connected to the maturation of B-cell specificity and the establishment of germinal centers [125,126,127], and its transcription is activated by IKAROS. In pre-B-ALL, chronic myeloid leukemia, and Ph-positive ALL cells, it performs as a tumor suppressor, controls the pre-BCR checkpoint and promotes apoptosis in response to oxidative stress [128,129,130]. In the case of IKZF1 deletion, BACH2 expression levels drop, resulting in a lower disease-free survival in pediatric ALL patients [108]. Ph+ ALL is characterized by an impaired pre-BCR function, and the loss of *IKZF1* activity increases SRC phosphorylation, impeding activation of the SYK/SLP65 pathway, which is necessary for pre-B-cell differentiation [109]. IKAROS involvement in pre-B-cell differentiation is well known [41,50,59]. Consequently, gene deletion involving the lymphoid transcription factors, such as *EBF1*, *PAX5* and *BTG1,* combined with the mono-allelic *IKZF1* deletion, may cause a remarkable blockage of B-cell growth and amplified proliferative spread of precursor B cells [73,85]. In ALL, the recombination process driven by RAG is not only the predominant variant process but also the predominant driver of oncogenic genomic rearrangements. Specifically, *RAG1* is a direct target of IKAROS, and its upregulation is considered a cell proliferation marker in B-ALL. Therefore, one of the oncogenic pathways that drives oncogenesis in B-ALL may be amplified by both *RAG1* high expression and *IKZF1* deletion. A study by Han et al. showed that *RAG1* high expression is related with *IKZF1* deletion, since patients with *RAG1* high expression have a much higher detection rate of the IK-6 isoform [115]. 

### 5.3. Daily Cellular Occupations: Adhesion and Metabolism Activities

The expression of cell surface proteins with intracellular WNT and RHO signaling, as well as catenin-driven gene regulation inside the nucleus, appears to be connected in a cellular network by another set of IKAROS target genes discovered in mouse progenitor B cells. *CTNND1*, which encodes the protein p120-catenin, is an important target gene of this subgroup. In samples from patients with *IKZF1* deletions, *CTNND1* expression is seen to be activated [106], and inactivating p120-catenin lowers the ability of Ph+ leukemic cells to proliferate [110,131]. Integrin-dependent survival signaling, characterized by the activation of focal adhesion kinase (FAK), is a related downstream effector pathway of IKAROS and crucial for mouse B-cell development [41,47]. Accordingly, *IKZF1* disruption in Ph+ B-ALL mice models, including loss-of-function deletions and IK-6 expression, activates an adhesive phenotype correlated with FAK overexpression [92,107]. Additionally, FAK pathway amplification is seen in Ph+ BCP-ALL, particularly when IK-6 expression is present. Hence, cell adhesion pathways are reactivated when the IKZF1 function is disrupted, resulting in elevated levels of key adhesion molecules such as integrins (ITGA5) and CD90 as well as adhesion regulators such as FAK, and increased phosphorylation of FAK itself, allowing leukemic cells to be relocalized to the bone marrow niche [107]. 

According to a recent study, the B-lymphoid transcriptional program controlled by IKAROS may operate as a metabolic barrier to prevent the cancerous transformation of BCP cells [105]. Metabolic investigations by Chen et al. [105] showed that IKAROS, as well as PAX5, enforces a condition of chronic energy scarcity, which results in constitutive activation of the energy-stress sensor AMPK5 [132,133,134] and lower concentrations of the proteins encoding the insulin receptor, the glucose transporters GLUT1, GLUT3, and GLUT6, as well as glucose metabolism effectors such as HK2, HK3, and G6PD. On the other hand, IKAROS strongly induces the expression of glucose-transport inhibitors such as TXNIP and CNR2. However, in pre-B-ALL cells, this glucose and energy restriction was alleviated by dominant negative *IKZF1* mutants, allowing sufficient amounts of cellular ATP for malignant transformation [105]. 

### 5.4. Cell Chatting: Signal Transducer and Cell Surface Receptors

Furthermore, IKAROS regulates the expression of cell surface receptors, including CD34 and CD43, and these molecules give *IKZF1*-mutated Ph- B-ALL cells a leukemic growth advantage [110]. Many of those IKAROS target genes fall under the category of signal transducers, some of which, such as c-KIT, FLT3, and IL7R, promote early lymphoid differentiation [41,50,59,116]. In fact, high expression of FLT3, IL7R and c-KIT is strongly associated with the loss of IKAROS function [116]. Moreover, several data suggest that loss of IKAROS function closely collaborates with the triggering of tyrosine kinase signaling pathways associated with increased progenitor B-cell proliferation and immortalization [117]. IL7R regulates T- and B-cell development and is necessary for the differentiation of hematopoietic cells into lymphoid progenitor cells [135,136]. To form the IL7 receptor or the thymic stromal lymphopoietin (TSLP) receptor, the *IL7R* gene codes for the IL7R-α chain heterodimers with the IL7R-γ form or with the cytokine receptor-like factor 2 (CRLF2), respectively [137,138]. Tyrosine residues on the receptor are phosphorylated due to receptor coupling, activating downstream JAK/STAT and PI3K/AKT/mTOR signaling cascades [139,140]. In leukemia, gain-of-function variants have been found in both T-ALL and B-ALL, and somatic variants in the *IL7R* gene are common in 10% of pediatric T-ALL patients [118]. A variant of adult B-ALL with high *IL7R* expression and low *SH2B3* expression has recently been discovered, and has been linked to a more serious clinical presentation and a poor prognosis [119]. Further analysis of samples from adult ALL with *IKZF1* deletions, a known subset of high-risk (HR) B-ALL, exhibited elevated *IL7R* expression and decreased SH2B3 expression [120]. By accelerating STAT3 phosphorylation and increasing NOTCH-1 signaling, loss-of-function variants in *SH2B3* have been linked to the oncogenesis of myeloproliferative neoplasms (MPN), early T-ALL, Ph-like ALL, B-ALL, and non-malignant hematological disorders [141,142,143,144]. 

Overall, the studies reported above demonstrate that IKAROS acts as the main character in regulating IL7/JAK/STAT5 signaling, thus promptly controlling the transcription of *CRLF2*, *IL7R* and *SH2B3*, which are crucial parts of this signaling pathway [119]. Indeed, it has been amply demonstrated that 43% of pediatric ALL cases with overexpression of *CRLF2* have *IKZF1* deletions [121]. Several studies have shown that IKAROS regulates the expression of its target genes in ALL by chromatin remodeling [34,111,122]. The suppression of CRLF2 expression partially brings on IKAROS tumor-suppressive actions in ALL. Accordingly, IKAROS binds to the *CRLF2* promoter region and reduces its expression in ALL cells by changing the promoter’s epigenetic signature. In HR-ALL without *CRLF2* rearrangement, loss of *IKZF1* may contribute to higher *CRLF2* levels. Therefore, elevated *CRLF2* levels may work with *IKZF1* deletion to promote ALL oncogenesis [100]. 

### 5.5. DNA Makeover: Epigenetic Signaling

IKAROS is responsible for the downregulation of *KDM5B* by recruiting HDAC1 to the *KDM5B* gene promoter [34,145]. The histone lysine demethylase KDM5B is involved in tumor induction, infiltration, and metastasis [146] by controlling the methylation levels of H3K4 in cancer cells, influencing the expression of tumor suppressors and oncogenes [101,146]. In numerous cancers, it has been demonstrated to be mutated and overexpressed [101,102]. Specifically, *KDM5B* expression was shown to be higher in B-ALL cells than in healthy bone marrow. Experimental studies have demonstrated that *KDM5B* gene suppression was caused by overexpression of IKAROS in both B-ALL and T-ALL cells. In contrast, a higher expression of *KDM5B* was observed when *IKZF1* was knocked down [31]. Plant Homeodomain Finger 2 (*PHF2*) was identified as an direct IKAROS target by Ge et al. [103]. PHF2, a positive epigenetic modulator, has been associated with tumor suppression in several malignancy types. Different subsets of ALL patients exhibit a significantly lower *PHF2* expression, which is correlated with leukemic cell proliferation. *IKZF1* deletion and low PHF2 levels in combination are two potential indicators of HR-ALL. Patients with B-ALL with a single deleted copy of IKZF1 had decreased PHF2 levels [100,103]. The AT-rich interactive domain (ARID) family of DNA-binding factors is one of the families most often dysregulated across various malignancies [147,148]. One significant member of the ARID family of proteins is ARID5B, which is crucial for differentiating and expanding B-cell progenitors [149]. Studies have shown that HDACs and the histone demethylase PHF2 interact with ARID5B [150,151]. The IKAROS/PHF2/ARID5B axis activates gene transcription. Recent research examining the genome-wide association of *ARID5B* has demonstrated that SNPs within *ARID5B* are critically associated with HR B-ALL [152]. Moreover, *IKZF1* and *ARID5B* SNPs may be positively associated with ALL, according to numerous studies [153,154,155]. Considering that ARID5B expression is positively controlled by IKAROS, Ge et al. revealed that low expression of ARID5B is correlated with the loss of a single copy of *IKZF1* [104]. Overall, the data indicate that low levels of *ARID5B* expression are involved in the oncogenesis of HR-ALL, and that a subset of HR-ALL is defined by low *ARID5B* and *PHF2* expression as well as haploinsufficiency of *IKZF1*. 

Numerous cellular activities are regulated by dynamin 2 (DNM2), including intracellular vesicle production and trafficking, receptor endocytosis, actin–microtubule interactions, cytokinesis, cell invasion and migration, and apoptosis regulation [156]. It has been hypothesized that DNM2 is crucial for the internalization of TCR, IL7R, and the NOTCH ligand Delta like 1 (DIl-1), which leads to the onset of ALL [157]. The link between IKAROS and DNM2 has been detected in B-ALL and T-ALL cells [158] as IKAROS binds its promoter, repressing *DNM2* and inducing the formation of heterochromatin. A reduced transcription of *DNM2* was caused by the overexpression of IKAROS in both kinds of leukemia. This was linked to an enrichment of the epigenetic marker H3K9me3 at the *DNM2* promoter. Meanwhile, the expression of DNM2 was enhanced by *IKZF1* knockdown [158]. 

### 5.6. IKAROS Remodelling: IKAROS/CK2/PP1 Axis 

Loss of function of *IKZF1* can potentially be caused by post-translational changes in addition to expression of the DN isoforms. Consequently, pathways that regulate IKAROS functions are involved in the leukemogenesis process. As regards the IKAROS/CK2/PP1 axis, there has been found to be a direct functional connection between IKAROS phosphorylation and signaling pathways. Its inability to undergo dephosphorylation by PP1 leads to its hyperphosphorylation by CK2, loss of DNA-binding capacity, localization in the pericentromeric region, as well as enhanced destruction by the ubiquitin pathway [23]. These findings demonstrate that two opposing signal transduction pathways, the tumor suppressor PP1 pathway and the oncogenic CK2 pathway, converge on IKAROS and exercise their oncogenic or tumor suppressor effects by controlling its function [159]. Multiple malignancies, including leukemia, overexpress the multipotent serine/threonine kinase CK2 [160]. When CK2 is overexpressed in B-ALL, it inhibits IKAROS complex formation and attracts HDAC1 to the *BCL2L1* promoter, which represses *BCL2L1* and promotes BCL-XL expression [161]. In the HR model of xenotransplantation in patients with ALL, pharmacological inhibition of CK2 can restore the DNA-binding affinity and tumor inhibitory activity of IKAROS and cause leukemia cytotoxicity, demonstrating the possibility of using CK2 inhibitors as therapeutic approaches for HR pediatric leukemia [122,162]. 

Accordingly, *IKZF1* gene deletions and variants in ALL patients impair a number of processes, including pre-BCR signaling, cell adhesion, proliferation and epigenetic signaling, metabolic and B/T-cell generation pathways, signal transduction, and cell surface receptor signaling. 

## 6. IKAROS in AML: A “Tweet” of Interest

IKAROS has a well-established role in lymphoid differentiation and ALL, but its function in myeloid differentiation is unclear. However, according to some compelling evidence, it also appears to have a role in myeloid differentiation. Notwithstanding several findings evidencing *IKZF1* involvement in the suppression of myeloid differentiation [163], its real role is unclear, and its alterations in AML are less studied. Despite this, *IKZF1* seems to take part in erythropoiesis, promoting erythrocyte differentiation at the expense of granulocyte and monocyte differentiation and supporting the survival of the erythroid lineage [164]. In addition, loss of IKZF1 function in early myeloid progenitors prolongs cell survival [165]. In early and late megakaryopoiesis, IKAROS regulates the transcription of genes involved in the NOTCH pathway as well as transcription factor genes such as *GATA1* and *RUNX1* [30,66]. With regard to granulocytes and monocytes, IKAROS represses the basophilic granulocyte lineage’s differentiation and promotes early maturation and survival of the neutrophil granulocyte lineage [20,21,164]. In detail, IKAROS is highly expressed in early myeloid precursor cells, and it is clear that the suppression of myeloid differentiation is as important as the promotion of lymphoid development [7]. 

### 6.1. IKZF1: An Emerging Character in the Pediatric AML Scenario

Despite this, not much is known about IKAROS’ involvement in AML. Several studies have suggested that protein kinase CK2 is implicated in the AML pathogenesis. In AML, protein kinase CK2 is active and encourages cell survival and resistance to apoptosis [98,166]. Notably, AML leukemia stem cells (LSCs) (CD34+ CD38- LSC) that have high levels of CK2 kinase activity are associated with poor patient outcomes. Thereby, IKAROS is phosphorylated and inactivated by a hyperactive CK2 system, contributing to AML treatment resistance. However, IKAROS suppresses the LSC ability to proliferate by repressing BCL-XL transcription, acting as a tumor suppressor in AML [98]. *IKZF1*-null animals exhibit hematopoietic stem cell abnormalities, including a decrease in myeloid cells, in addition to a complete absence of B-cells, dendritic cells, and their progenitors. 

Supporting this, it has been reported that a child with a constitutional, de novo heterozygous point variant in the IKZF1 gene exhibited congenital pancytopenia similar to the hematopoietic abnormalities seen in the IKZF1-null mouse model [167]. This fact suggests that IKAROS is a crucial regulator of normal hematopoiesis. Considering its involvement in early myeloid precursor cells, it is plausible that reduced IKAROS expression during these stages increases the susceptibility to infant AML. However, Ross et al. [163] showed, by genotyping 450 AML cases (ages 0–19 years), that no overall associations with the variant allele were found, suggesting that this susceptibility is specific to infants and is not a feature of AML generally. 

In pediatric AML, monosomy 7 is a recurrent chromosome loss; in particular, in myeloproliferative diseases that have progressed towards AML (40% of cases), but also in primary AML (4–5% of cases) [168] (Figure 2B). However, the 5-year OS and EFS of pediatric AML patients with monosomy 7, with or without additional cytogenetic or chromosomal aberrations, are poor [168]. De Rooij et al. demonstrated several *IKZF1* alterations in a cohort of AML child patients, such as focal deletion, single nucleotide variation leading to amino acid changes and different cases of monosomy 7 [169]. Notably, the gene profile of AML patients with monosomy 7 and those with focal deletion of *IKZF1* are similar; not only that, patients with monosomy 7 have a worse outcome than those with a 7p deletion [168]. 

### 6.2. IKZF1 Loss in AML: Is It Only a Consequence of Cytogenetic Alterations?

In adult AML, monosomy 7 is also the most frequent single monosomy [170]. Meanwhile, deletions of the short arm of chromosome 7 (del(7p)) are recurrently found in adult de novo and in secondary AML developing from myelodysplastic syndrome (MDS) or myeloproliferative neoplasms (MPN) [171,172]. Several studies have suggested that del(7p), resulting in loss of *IKZF1*, is a feature more frequently associated with secondary AML, while it is rare in de novo AML or chronic phase of MDS or MPN [171], indicating that *IKZF1* loss contributes to the transformation to AML. Zhang et al. have identified seven different variants of *IKZF1* [3]. Interestingly, *IKZF1* variants were absent in the fusion gene-positive AML cohort reported by Zhang et al. Analysis of the frequency of *IKZF1* variants in three independent studies has evidenced that IKAROS^N195S^ can be considered a hotspot variant in AML, which could be defined as one independent subtype that remains to be investigated [173,174]. The latest study by Zhang et al. confirmed that an *IKZF1* variant is a rare event in the AML context, and its frequency was low (4.15%) in their newly diagnosed AML cohort [175]. Moreover, a growing body of evidence suggests that *IKZF1* disruption also affects the myeloid hierarchy [28,176]. Together, these data show that *IKZF1* may have a role in myeloid differentiation; moreover, the *IKZF1* loss may contribute to myeloid oncogenesis, although its functional consequences require further investigation.

## 7. Conclusions

In conclusion, we delineate a “social” profile of IKAROS, a decisive transcription factor that regulates the expression of several genes in normal hematopoietic cells and leukemia. IKAROS target genes are involved in various cellular processes, such as pre-BCR signaling (e.g., *BACH2*), cell adhesion (e.g., *FAK*), proliferation (e.g., *PI3K*, *c-MYC*) and epigenetic signaling (e.g., *KDM5B*, *PHF2*, *ARID5B*), metabolic (e.g., *AMPK5*) and B/T-cells differentiation pathways (e.g., *RAG1/2*), signal transduction (e.g., c-*KIT*, *IL7R*, *CLRF2*), and cell surface receptor signaling (e.g., *CD34*, *CD43*). All these pathways are undoubtedly affected in the context of ALL, but several emerging studies suggest possible alterations also in AML. IKAROS alterations are well known in ALL patients; the most frequent are deletions of the entire gene or part of it [11]. Meanwhile, in AML, several *IKZF1* variants have recently been reported, including point variants [175]. Considering that monosomy 7 is a recurrent chromosome loss in AML, *IKZF1* haploinsufficiency may have a pivotal role in leukemogenesis, although this has yet to be clarified. In view of its ambivalent role as a tumor suppressor and oncogenic factor and the numerous alterations affecting this role, IKAROS can be considered a possible key driver in the pathogenesis of ALL as well as AML. The discovery of additional IKAROS target genes is crucial to learn more about IKAROS’ role as a tumor suppressor and identify additional therapeutic targets for AL. Because of the intricate network of pathways in which it is involved, further studies of IKAROS may reveal its real contribution also in AML leukemogenesis. Several topics need to be addressed in the future, such as the different clinical impacts of the several alterations that affect the *IKZF1* gene and the corresponding IKAROS protein in AL patients. Additionally, further investigation of cooperative genetic lesions that may alter the effect of *IKZF1* defects is necessary to obtain accurate information that can then be used in clinical decision making.

## Data Availability

Not applicable.
